# *In Vitro* HIV Infection Impairs the Capacity of Myeloid Dendritic Cells to Induce Regulatory T Cells

**DOI:** 10.1371/journal.pone.0042802

**Published:** 2012-08-13

**Authors:** Pietro Presicce, Maria E. Moreno-Fernandez, Laura K. Rusie, Carl Fichtenbaum, Claire A. Chougnet

**Affiliations:** 1 Division of Cellular and Molecular Immunology, Cincinnati Children’s Hospital Research Foundation, and Department of Pediatrics, University of Cincinnati College of Medicine, Cincinnati, Ohio, United States of America; 2 Infectious Disease Center, University of Cincinnati College of Medicine, Cincinnati, Ohio, United States of America; New York University, United States of America

## Abstract

Myeloid dendritic cells (mDCs) are the antigen-presenting cells best capable of promoting peripheral induction of regulatory T cells (Tregs), and are among the first targets of HIV. It is thus important to understand whether HIV alters their capacity to promote Treg conversion. Monocyte-derived DCs (moDCs) from uninfected donors induced a Treg phenotype (CD25^+^FOXP3^+^) in autologous conventional T cells. These converted FOXP3^+^ cells suppressed the proliferation of responder T cells similarly to circulating Tregs. In contrast, the capacity of moDCs to induce CD25 or FOXP3 was severely impaired by their *in vitro* infection with CCR5-utilizing virus. MoDC exposure to inactivated HIV was sufficient to impair FOXP3 induction. This DC defect was not dependent on IL-10, TGF-β or other soluble factors, but was due to preferential killing of Tregs by HIV-exposed/infected moDCs, through a caspase-dependent pathway. Importantly, similar results were obtained with circulating primary myeloid DCs. Upon infection *in vitro*, these mDCs also killed Treg through mechanisms at least partially caspase-dependent, leading to a significantly lower proportion of induced Tregs. Taken together, our data suggest that Treg induction may be defective when DCs are exposed to high levels of virus, such as during the acute phase of infection or in AIDS patients.

## Introduction

Dendritic cells (DCs) are the most potent antigen presenting cells (APCs), endowed with the unique ability to prime naïve CD4^+^ T cells. They are believed to be an important target for HIV during sexual transmission due to their presence at mucosal surfaces, their function as antigen capturing cells and their role in initiating adaptive immune responses [1]. DCs can be directly infected by HIV (*cis*-infection), although the frequency of *in vivo* infected DCs is 10- to 100-fold lower than that of infected CD4^+^ T cells [2]. However, DCs can also transmit virus to CD4^+^ T cells (*trans*-infection), without being productively infected [3], [4]. HIV induces a semiactivated phenotype in DCs and compromises their functionality by impairing cytokine production and antigen presentation [1]. *In vitro*, myeloid dendritic cells (mDCs) are more susceptible to R5 HIV (virus using CCR5 as co-receptor) infection than plasmacytoid DCs (pDCs) due to their higher expression of CCR5 [5]. Due to the low frequency of circulating mDCs, monocyte-derived dendritic cells (moDCs) are often used as a model of mDCs. MoDCs can be infected by HIV similarly to infected mDCs [5]. MoDCs mature when exposed to lipopolysaccharide (LPS), which increases their ability to mediate HIV *trans*-infection [3].

Regulatory T cells (Tregs) are a subset of CD4^+^ T cells critical to maintenance of immunological self-tolerance and immune homeostasis [6]. The role that Tregs play in the context of chronic infection such as HIV remains unclear. Some authors have reported that *in vitro* removal of Tregs from HIV-infected humans and SIV-infected macaques enhances antiviral immune responses [7], [8], and it has been proposed that excessive Treg reactivity suppresses the function of multiple cell types and leads to faster progression of HIV pathogenesis [8]. On the other hand, Tregs may protect individuals from the deleterious effects of immune activation that are typically observed in chronic infection (reviewed in [9]).

Treg frequency increases in lymphoid tissues as well as peripheral blood during chronic HIV infection [10]–[15], but the underlying mechanisms have not yet been characterized. In addition to natural Tregs that arise and mature in the thymus, growing evidence demonstrates that Tregs can be induced from either naïve (reviewed in [16]) or memory [17] conventional CD4^+^ T cells in the periphery. Recent data highlight the role of APCs, mDCs in particular, in inducing FOXP3 expression and suppressive function in conventional CD4^+^ T cells [18], [19]. Manches et al. reported that pDCs exposed *in vitro* to HIV induced Tregs from allogeneic naïve CD4^+^ T cells *via* an indoleamine 2,3-dioxygenase (IDO)-dependent mechanism [20]. Recently, we showed that tissue mDCs induce the conversion of nonTregs into Tregs in chronically SIV-infected macaques [21]. Thus, mDC-mediated conversion may contribute to the accumulation of Tregs observed in lymphoid tissues of HIV/SIV-infected subjects [10], [11], [13], [22]. To investigate whether DCs in circulating blood could mediate the accumulation of Tregs in acute HIV infection, we examined whether *in vitro* infected moDCs as well as primary mDCs from healthy donors could convert autologous nonTregs into Tregs and whether these converted cells were functional.

Surprisingly, our results show that *in vitro* infected DCs were severely impaired in their capability to induce functional Tregs, and that this defect was mainly associated with the increased death of T cells cultured with the infected DCs. This death was contact-dependent and at least partially caspase-mediated. Importantly, similar results were obtained with primary mDCs infected *in vitro*. Exposure of DC to HIV was sufficient to alter their function. Collectively, our data suggest that Treg induction by DCs may be defective when they are exposed to high levels of virus, such as during the acute stage of infection or AIDS.

## Materials and Methods

### Cell Isolation and Culture

Peripheral blood mononuclear cells (PBMCs) were separated by centrifugation through Ficoll–Hypaque (GE, Fairfield, CT). CD14^+^ monocytes were isolated by positive selection (CD14 beads, Miltenyi Biotec, Auburn, CA) and immature moDCs were generated by culturing the isolated monocytes for 5 days in complete medium (RPMI 1640, supplemented with 10% of heat-inactivated fetal calf-serum, HEPES, Glutamine) with 500 U/ml rhIL-4 (Peprotech Inc, Rocky Hill, NJ) and 1000 U/ml GM-CSF (Peprotech). One-third of complete medium, including cytokines, was replaced every 3 days. For some experiments primary mDCs were purified from circulating elutriated monocyte fraction on a MoFlo XDP Cell Sorter (Beckman Coulter, Brea, CA). Cells were stained in PBS containing 2% FBS using fluorochrome-conjugated antibodies and mDCs were defined as CD14^−^HLA-DR^+^CD11c^+^ with purity >98% [23]. MoDCs as well as mDCs were stimulated overnight with 500 ng/ml of LPS (Sigma-Aldrich). Immature moDCs expressed low levels of activation/maturation markers (CD80, CD86, CD40, PDL-1, HLA-DR and CD83), while LPS-mature moDCs expressed high levels of these markers.

Resting autologous CD4^+^ T cells were first purified from the CD14^−^ cell population by negative selection (CD4 separation kit, Miltenyi Biotec), as described above. Tregs and nonTregs were further separated by cell sorting using a FACSAria (BD), with Tregs defined as CD8^neg^CD25^hi^CD127^low^ and nonTregs defined as CD8^neg^CD25^low^CD127^hi^. In some experiments, nonTregs were further separated into naïve defined as CD8^neg^CD25^low^CD127^hi^CD45RA^pos^ and memory defined as CD8^neg^CD25^low^CD127^hi^CD45RA^neg^. The purity of Tregs and nonTregs was evaluated post-sorting by intracellular detection of FOXP3 (clone PCH101, e-Bioscience, San Diego, CA). Purified Tregs were >90% FOXP3^+^, whereas nonTregs were less than 0.5% FOXP3^+^. Where indicated, CD25^−^ nonTregs (<1.3% FOXP3^+^ cells post-isolation) and CD25^+^ Tregs (>80% FOXP3^+^ cells post-isolation) were purified from CD4^+^ T cells using CD25 magnetic beads (Miltenyi Biotech).

### Treg Phenotypic Characterization

The following antibodies (Abs) were used for phenotypic characterization of induced Tregs and DCs. Anti-CD14 (61D3) FITC-, anti-CD1a (HI149) PE-, anti-CD40 (5C3) FITC-, anti-CD86 (FUN-1) FITC-, anti-HLA-DR (L243) PerCP-, anti-CD11c (3.9) APC-, anti-CD83 (HB15e) APC-, anti-CD80 (2D10) PE-, anti-PDL-1 (MIH1) PE-, anti-CD3 (SK7) PerCPCy5.5-, anti-CD4 (RPA-T4) PB-conjugated were purchased from BD Biosciences (San Diego CA). Anti-FOXP3 (PCH101) PB- and AF647-conjugated were obtained from eBioscience. Anti-CTLA-4 (14D3) PE-, anti CD45RA (MEM-56) PE-Cy7-, anti-CD45RO (UCHL-1) PB, anti-CD25 (M-A251) APC-H7-conjugated were purchased from BD Biosciences (San Diego, CA). Anti-CD127 (R34.34) PE-conjugated was purchased from Beckman Coulter (Fullerton, CA). Anti-CD4 (RPA-T4) AF700-conjugated was purchased by Biolegend (San Diego, CA). Unconjugated anti-GARP (Plato-1) was purchased from Alexis Biochemicals (San Diego, CA) and conjugated using Zenon PE mIgG2b labeling kit (Invitrogen; Carlsbad, CA) following manufacturer’s instructions. All antibodies were titrated for optimal detection of positive populations and mean fluorescent intensity (MFI) prior to use.

Cells were treated with 20 µg/ml of human IgG to block Fc receptors and stained for surface markers for 30 min at 4°C, in PBS containing 2% fetal calf serum and 0.1% sodium azide. Cells were then washed and fixed with Fixation/Permeabilization Buffer (eBioscience). After 30 minutes of incubation at 4°C, cells were washed and permeabilized with Permeabilization Buffer (eBioscience) and incubated with rat serum for 15 min at 4°C to block non-specific binding of antibodies. Cells were finally stained with FOXP3 and CTLA-4 mAbs for 30 min at 4°C and analyzed on FACS LSR-II (BD). At least 150,000 events were recorded for each sample. Doublets were excluded on the basis of forward- and side-scatter properties and dead cells were gated out using LIVE/DEAD Viability kit (Invitrogen). Data were analyzed using FACSDiva (BD) and FlowJo software (TreeStar Inc., Ashland, OR, USA).

### Virus Production and DC Infection

Simian Immunodeficiency Virus (SIV)mac-Virion Like-Particles (VLPs) and HIV viruses were prepared by transfection of 293 T cells with plasmids encoding the R5-tropic (YK-JRCSF) HIV lab strain and SIV-mac VLP, using FuGENE (Roche) [24]. After two days, supernatants were harvested, and the viruses were precipitated using polyethyleneglycol. YK-JRCSF virus titer was determined using TZM-bl indicator cells as previously described [25]. SIVmac-VLPs titers were determined using the Reverse Transcriptase colorimetric assay kit (Roche). In addition, for some experiments, YK-JRCSF-containing supernatants were treated with 1 mM 2,2′-aldrithiol (AT-2) for 1 hour at 37°C in agitation, as previously described [26]. This treatment has been shown to eliminate infectivity of retroviral virions while preserving the conformation of envelope glycoproteins. Microvesicles, isolated from uninfected cultures of 293 T cells following the same procedures as those used to prepare AT-2 HIV, were included in the experiments as negative control.

LPS-mature DCs were infected at different multiplicities of infection (MOI, from 0.01 to 3). Briefly, DCs were incubated with YK-JRCSF plus SIVMAC-VLPs for 6 hours at 37°C, washed twice with complete RPMI, and cultured for 24 hours [26] before culture with nonTregs. For some experiments DCs were treated with Zidovudine (AZT, 1 µM) at the time of HIV infection and during the co-cultures.

To quantify integrated HIV DNA in DCs, infection of LPS-matured moDCs was carried out with viruses pre-treated with DNase I at 20 U/ml in 10 mM MgCl_2_ to eliminate cellular DNA carryover from virus production. After 18 hours at 37°C, cells were collected and suspended in lysis buffer containing 10 mM Tris HCl (pH 9), 0.1% Tween 20-NP40 and 400 µg/ml Proteinase K (Invitrogen). Cellular lysates were used to quantify cell-associated HIV-1 DNA by nested real-time PCR [27]. Briefly, the first round of amplification used *Alu* specific primers and the HIV-1 long terminal repeat (LTR). In the same reaction, the CD3 gene was quantified to precisely determine the number of input cells. This reaction was followed by a second round of amplification with specific primers and a labeled probe specific for the HIV LTR, performed in a Light Cycler (Roche). In parallel, CD3 was re-amplified and detected using SYBR Green. The ACH-2 cell line, a line of human T-lymphocytic leukemia that contains a single copy of HIV-1 proviral DNA, was used to determine the efficiency of the primers (NIH AIDS Research and Reference Reagent Program) (detection limit = 3 copies of HIV DNA).

### Treg Suppression Assays

Cells from nonTreg/DC co-cultures (hereafter referred to as “bulk”, containing converted Tregs) were harvested after 5 days of culture and mixed with autologous purified CD4^+^CD25^low^CD127^hi^ nonTregs (i.e. responder cells) at a 10∶1 ratio in presence of immobilized anti-CD3 Ab (96 well U-bottom plates were coated with 1 µg/ml Ab, BD Biosciences, at 37°C for 2 hours) and soluble anti-CD28 Ab (0.5 µg/ml, BD Biosciences). Responder nonTregs were CFSE-labeled before stimulation. In one well, responder cells were cultured alone as negative control. As positive control, purified Tregs (CD4^+^CD25^hi^CD127^low^) were mixed with autologous nonTregs at different ratios (0.1∶1, 0.5∶1, 1∶1, 1∶2, 1∶4). After 4 days, cells were harvested and the suppression ability of Tregs was determined by analyzing the percentage of responder cells having divided at least once (CFSE^low^) in the different cultures.

### *In vitro* Modulation of Treg Induction

Recombinant human IL-10 (rIL10) was purchased from Peprotech (Rocky Hill, NJ) and used at 10 ng/ml (after titration). Recombinant human TGF-β (rTGF-β) and rPDL-1 were purchased from R&D Systems (Minneapolis, USA) and used at 10 ng/ml (after titration). Neutralizing antibodies against human IFN- γ-receptor 1 (IFN-γR1), IL-4R (R&D Systems), IL-10 and IL-17 (BD Bioscences) were used at 10 µg/ml, while neutralizing antibodies against human TGF-β (Sigma Aldrich) was used at 10 ng/ml. Recombinant IL-2 (rIL-2) was used at 100–1000 IU/ml. All the above mentioned reagents were added at the beginning of the co-cultures. The pan-caspase inhibitor Z-VAD-FMK was purchased from R&D Systems and added twice daily starting at day 0 at a final concentration of 50 µM.

### TGF-β Detection

Cellular RNA was isolated using TRIZOL. TGF-β and Ubiquitin ligase (UBI) mRNA were quantified by RT-PCR using the Light Cycler (Roche). All reactions were performed in duplicate using a SYBR green PCR mix (Qiagen, Valencia, CA), according to the following thermal profile: denaturation at 95°C for 15 seconds, annealing at 60°C for 15 seconds, and extension at 72°C for 15 seconds (data collection was performed during the extension step). The following primers were used: TGF-β (QT00000728, Qiagen, Santa Clarita, CA) and UBI forward (QT02306724, Qiagen). The threshold level was determined by the software according to the optimization of the baseline and the standard curve. Results are presented as ratios between the target gene mRNA and the UBI mRNA.

### Statistical Analysis

Statistical analysis was performed using Prism (GraphPad Software 5). Paired *t-*test was used in the comparison between different conditions. Linear regression analysis was used to test the correlation between percentage of death in CD3^+^CD4^+^ T cells and percentage of induced CD25^+^FOXP3^+^ Tregs as well as the correlation between percentage of memory cells and percentage of induced CD25^+^FOXP3^+^ Tregs, age and percentage of induced CD25^+^FOXP3^+^ Tregs. A p value of less or equal to 0.05 was considered to be significant.

## Results

### Uninfected Mature moDCs Induce Tregs but their Infection with HIV Impairs this Process

**Figure 1:**
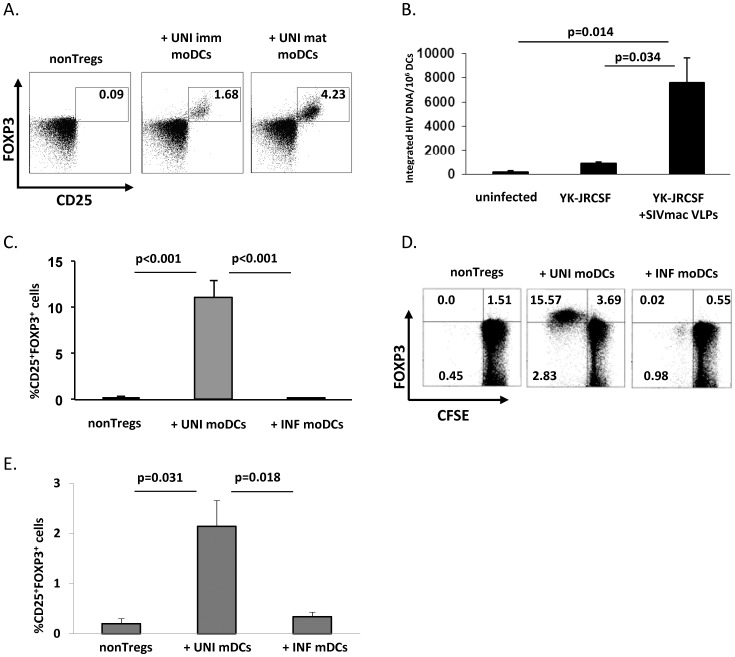
Infection of DCs by HIV blocks their induction of CD25 and FOXP3 in autologous nonTregs. (A) moDCs differentiated from purified CD14^+^ monocytes were stimulated with or without LPS (500 ng/ml). After 24 h, immature or LPS-mature moDCs were co-cultured with autologous purified nonTregs (CD4^+^CD25^low^CD127^hi^) for 5 days, at a DC: nonTregs ratio of 1∶10. As a control, nonTregs were cultured alone. A representative flow cytometry data comparing Treg induction by mature or immature moDCs is shown. B) moDCs differentiated as described in A) were stimulated with LPS (500 ng/ml) for 24 h. They were then infected with HIV YK-JRCSF, in presence or not of SIVmac VLPs (both at a MOI = 3). HIV proviral DNA levels were measured by nested real-time HIV-LTR-Alu PCR 24 h postinfection; HIV DNA levels were normalized based on CD3 quantification (n = 5). C) LPS-activated moDC were infected with HIV YK-JRCSF and SIVmac VLP (INF) or left uninfected (UNI), and co-cultured with autologous purified nonTregs (CD4^+^CD25^low^CD127^hi^) for 5 days, as described above. Mean ± SE % of CD25^+^FOXP3^+^CD4^+^ T cells (n = 18) is shown and one representative flow cytometry experiment showing CFSE levels in induced FOXP3^+^ cells is shown in D). E) Elutriated fraction of PBMC was stained with a cocktail of mAbs and mDCs were isolated based on CD14^−^HLA-DR^+^CD123^−^CD11c^+^ expression. After overnight stimulation with LPS, these cells were infected with HIV and cultured with autologous purified CD4^+^CD25^low^CD127^hi^ nonTregs for 5 days, as described above. Mean ± SE % of CD25^+^FOXP3^+^CD4^+^ T cells (n = 5) is shown.

Myeloid DCs are reported to be better inducers of Tregs than other APCs [19]. As DCs can be infected with HIV, we investigated the impact of their infection on Treg conversion. To this end, we compared the ability of *in vitro* infected moDCs and uninfected moDCs to induce CD25 and FOXP3 expression in purified nonTregs (defined as CD4^+^CD25^low^CD127^hi^ T cells, which contained less than 0.5% FOXP3^+^ cells). The optimal ratio of DCs to T cells for the FOXP3 induction was 1∶10 (0.05×10^6^ DCs: 0.5×10^6^ nonTregs), as determined in preliminary experiments using either autologous or allogeneic culture conditions. In addition, kinetic experiments showed that FOXP3 induction peaked after 5 days of culture. These experimental conditions were thus used throughout the study. LPS-activated moDCs induced higher expression of FOXP3 in nonTregs compared to unstimulated immature moDCs (Figure 1A). This increase was not specific to LPS stimulation as *Staphylococcus aureus* Cowan-activated moDCs induced similar percentages of FOXP3^+^ cells as LPS-moDCs (data not shown).

To achieve high levels of DC infection, LPS-activated moDCs (mature moDCs) were infected with the R5 HIV strain YK-JRCSF in presence of non-infectious virion-like particles derived from the Simian Immunodeficiency Virus (SIVmac VLPs) (both at 3 MOI). This method has been shown to increase the efficiency of transduction by HIV-1 lentiviral vectors. The effect is restricted to DCs and likely mediated by providing additional viral accessory protein Vpx that counteracts the restriction factor SAMHD1 [24], [28]. Consistent with these previous reports, addition of SIVmac VLPs significantly increased the levels of DC infection compared to infection with YK-JRCSF alone (Figure 1B). Notably, a similar viability >88% was observed in both uninfected and infected DCs.

Induction of both CD25 and FOXP3 expression was severely impaired when nonTregs were cultivated with *in vitro* HIV-infected moDCs (mean ± SE % of CD25^+^FOXP3^+^ cells: 10.02%±1.64% vs. 0.14%±0.05% in uninfected vs. infected co-cultures, respectively; p<0.001, n = 18, Figure 1C). Notably, LPS stimulation after infection did not affect the ability of DC to induce Tregs (not shown). Analysis of CFSE dilution indicated that FOXP3 expression appeared early after activation and persisted, as the level of FOXP3 was found to be equal in T cells having divided once or several times (Figure 1D).

To exclude the possibility that moDCs could expand the few contaminating FOXP3^+^ cells rather than convert nonTregs into Tregs, mature moDCs were cultivated with autologous purified CD25^hi^CD127^low^ Tregs (>90% FOXP3). While purified by cell sorting Tregs completely lost FOXP3 expression when cultivated alone without any kind of stimulation, expression of FOXP3 was partially maintained when they were cultured with mature moDCs (Figure S1). However, they did not proliferate, suggesting that the contamination by these cells does not contribute significantly to the pool of cycling FOXP3^+^ cells seen in the DC : nonTregs co-cultures (Figure S1). We also confirmed that conversion was a mDC-dependent mechanism, as neither FOXP3 nor CD25 were induced when non-Tregs were cultured with LPS alone (Figure S2). SIVmac VLPs have been reported to not affect DC function and maturation [24]. We nevertheless confirmed that infection of DCs with SIVmac VLP alone did not compromise their ability to induce FOXP3 (Figure S3).

### HIV-infected Primary mDCs are Defective in their Capacity to Induce FOXP3

We next wanted to confirm data obtained with infected moDCs with primary infected DCs. Purified circulating myeloid DCs (mDCs) from healthy donors were infected, or not, with HIV *in vitro*, and then cultured with autologous nonTregs. Uninfected mDCs were more efficient at inducing a Treg phenotype in nonTregs compared to infected mDCs (mean±SE% of CD25^+^FOXP3^+^ cells: 2.14%±0.52% vs. 0.39%±0.09% in uninfected vs. infected co-cultures, respectively; p = 0.018, n = 5, Figure 1E), although they were less efficient than moDCs. However, Treg induction by uninfected, purified mDCs was comparable to that observed using tissue-derived mDCs in macaques [21]. As a similar pattern of Treg induction was found for mDCs and moDCs, the majority of experiments were performed with moDCs, due to the paucity of primary mDCs.

**Figure 2:**
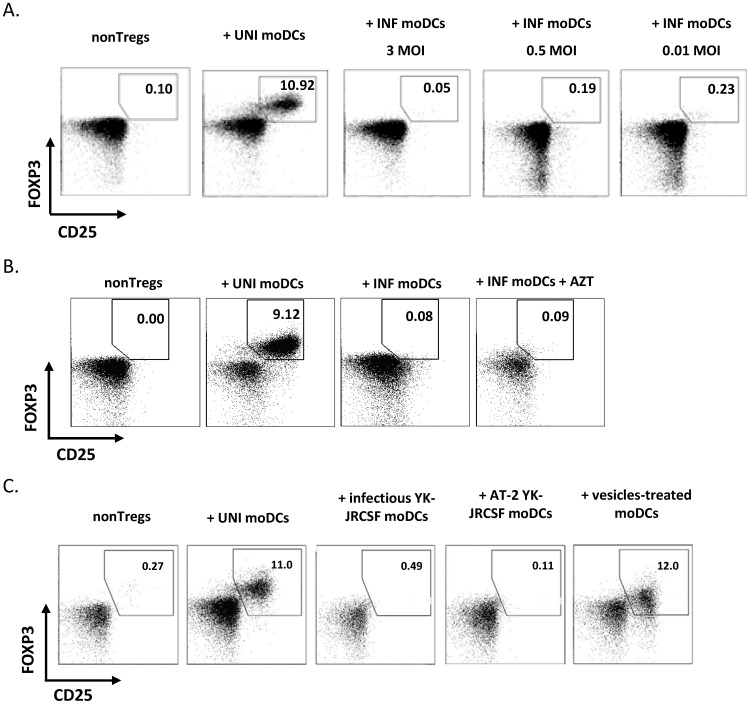
Exposure to HIV is sufficient to impair DC-mediated conversion by moDCs. A) Different MOIs of infectious YK-JRCSF were used to infect LPS-stimulated moDCs. Percentage of CD25^+^FOXP3^+^ T cells was analyzed after 5 days of co-culture. One representative example of 5 independent experiments is shown. B) In some experiments (n = 4), infected DC were treated or not with the reverse transcription inhibitor AZT (1 µM). Flow cytometry data from one representative experiment are shown. C) In some experiments (n = 4), LPS-activated moDCs were exposed to infectious or AT-2-treated YK-JRCSF+SIV VLPs. As a negative control, AT-2-treated microvesicles were used. Flow cytometry data from one representative experiment are shown.

### Exposure to HIV is Sufficient to Impair moDCs to Induce Tregs

Even low levels of HIV, as low as 0.01 MOI, abolished the ability of moDCs to induce Tregs (Figure 2A). To determine what stage of the HIV cycle affects DC function, we treated DCs with the reverse transcriptase inhibitor AZT before culture with nonTregs. This treatment did not restore Treg induction, suggesting that exposure to virus alone was sufficient to block DC-mediated Treg conversion (Figure 2B). To confirm these data, we inactivated HIV by treatment with AT-2, a mild oxidizing reagent that eliminates the infectivity of HIV while maintaining its structure and ability to be processed for presentation to T cells [29]. AT-2 YK-JRCSF-exposed moDCs were unable to induce Tregs, similar to moDCs infected with the replication-competent YK-JRCSF (Figure 2C). The addition of SIVmac VLP to AT-2 YK-JRCSF did not change these results (data not shown).

**Figure 3:**
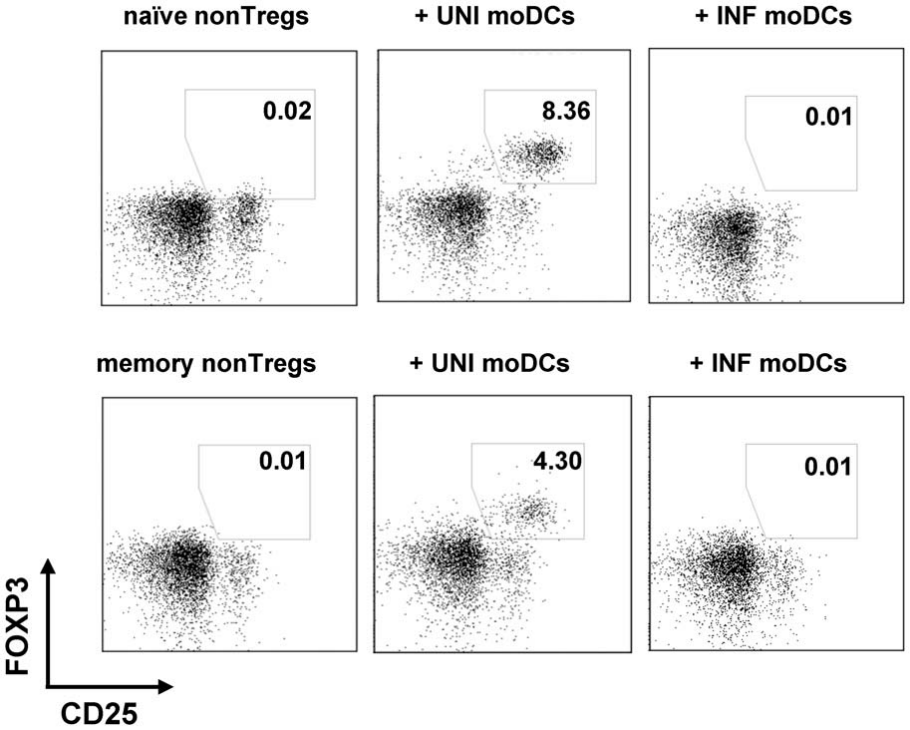
Naïve and memory nonTregs are converted into Tregs by uninfected moDCs. Uninfected or infected moDCs were cultured with purified naïve (CD4^+^CD25^low^CD127^hi^CD45RA^pos^, upper panel) and memory (CD4^+^CD25^low^CD127^hi^CD45RA^neg^, lower panel) nonTregs. The numbers in the quadrants represent the percentages of CD25^+^FOXP3^+^ induced Tregs. One representative example of 2 independent experiments is shown.

### moDCs Convert both Naïve and Memory nonTregs into Tregs

Many studies of *in vitro* Treg conversion have used naïve T cells, as murine and human memory T cells could not be converted to Tregs by *in vitro* stimulation with TGF-β. Furthermore, the production of cytokines by memory T cells can block the conversion of naïve CD4^+^ T cells into Tregs [30]. However, other studies suggest that memory human and murine nonTregs could also be converted into Tregs [17], [31]–[33], and DC-mediated conversion of human cells does not appear to be similarly blocked by inhibitory cytokines [19]. We nevertheless tested whether cytokines produced by memory T cells blocked Treg conversion by adding anti-IFN-γR1, anti-IL4R or anti-IL17 Abs to our cultures. FOXP3 induction was not increased by these antibodies (data not shown). Moreover, the efficiency of FOXP3 induction was not correlated with either the age of the donors (r = 0.08, p = 0.23) or the proportion of memory CD45RO^+^ T cells in purified nonTregs (r = 0.05, p = 0.39). To confirm these data, we purified naïve and memory nonTregs by cell sorting, and cultivated them with autologous uninfected or infected moDCs. DC-mediated conversion could occur in both nonTreg populations, although conversion was more efficient for naïve nonTregs than for memory nonTregs (Figure 3). Notably, the pattern of Treg induction in unfractionated nonTregs (containing both naïve and memory) was similar to that observed in purified naïve or memory nonTregs.

**Figure 4:**
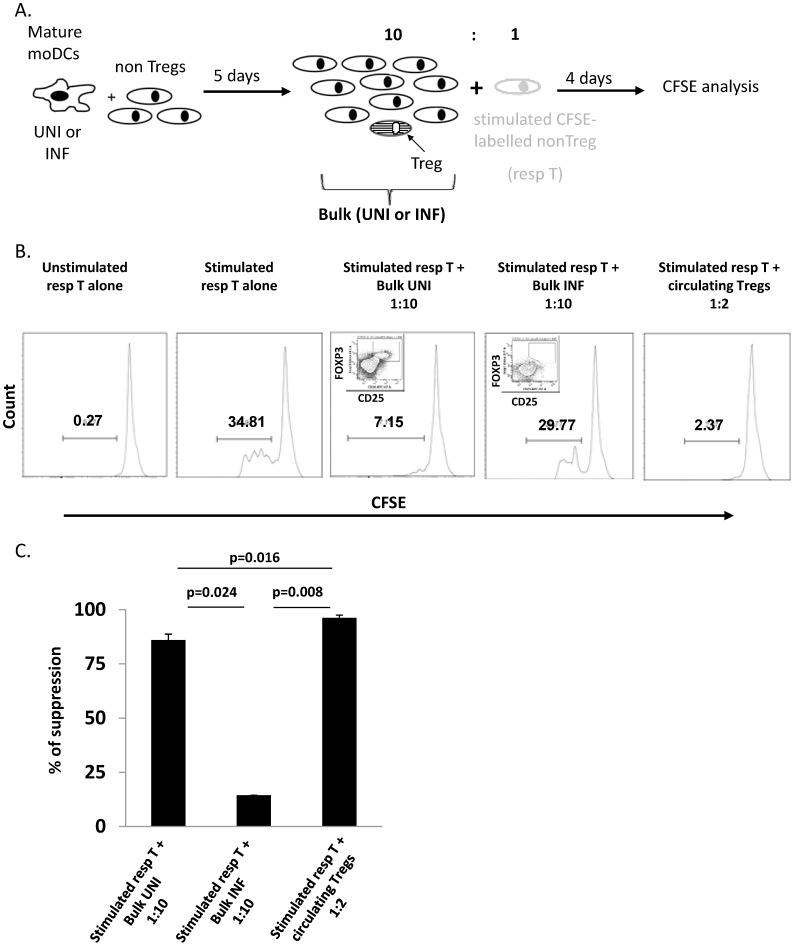
Induced Tregs suppress proliferation of responder T cells similarly to natural Tregs. Ability of induced CD4^+^FOXP3^+^CD25^+^ to suppress the proliferation of autologous responder nonTregs was assessed. A) Scheme showing the experiment design. NonTregs were cultured for 5 days with autologous mature moDCs, infected (INF) or not (UNI). After 5 days, these T cells (“bulk”) were mixed with CFSE-labelled autologous nonTregs (resp T), and stimulated by immobilized anti-CD3 Ab (1 µg/ml) and soluble anti-CD28 Ab (0.5 µg/ml) for 4 days. As the frequency of FOXP3^+^ cells within co-cultures containing uninfected DCs is close to 10%, we used a “bulk”: nonTregs ratio of 1∶10. At day 4, proliferation of responder nonTregs was determined by flow cytometric analysis of CFSE levels. Responder nonTregs were also cultured alone or were stimulated alone. As a control, circulating purified Tregs were added to responder nonTregs at a 2∶1 ratio. B) One representative example of 3 independent experiments is shown. Numbers in each panel represent the percentage of responder cells undergoing proliferation (CFSE low). The small inserts show the proportion of induced Tregs in the same experiment. C) Graph shows the mean ± SE % of suppression mediated by converted Tregs or circulating Tregs compared to proliferation of stimulated nonTregs alone (n = 3, paired t-test).

### Converted Tregs Suppress Proliferation of Autologous nonTregs, Similar to Circulating Tregs

FOXP3 expression can be transiently upregulated upon T cell activation, but does not always confer regulatory properties [34]. It was therefore important to test the functionality of the CD25^+^FOXP3^+^ Tregs generated by culture with moDCs. The intracellular localization of the transcription factor FOXP3 prevents sorting of live converted CD25^+^FOXP3^+^ T cells. For this reason, day 5 co-cultures containing both DCs and the converted FOXP3^+^ cells, were added in bulk to autologous stimulated nonTreg responder cells at a 10∶1 ratio (experimental scheme is detailed in Figure 4A). This ratio was chosen because nonTregs cultured with uninfected moDCs contained about 10% FOXP3^+^ cells (Figure 1C), giving an approximate ratio of 1 Treg : 1 responder cell. Importantly, cultures containing *de novo* induced FOXP3^+^ cells suppressed the proliferation of autologous nonTregs (Figure 4B). In contrast, nonTregs cultivated with infected DCs, which contained less than 1% of FOXP3^+^ cells, did not suppress the proliferation of responder nonTregs (Figure 4B). These data strongly suggest that the FOXP3^+^ cells induced *in vitro* are suppressive. Furthermore, the induced Treg were as suppressive as circulating Tregs (Figure 4B and 4C).

**Figure 5:**
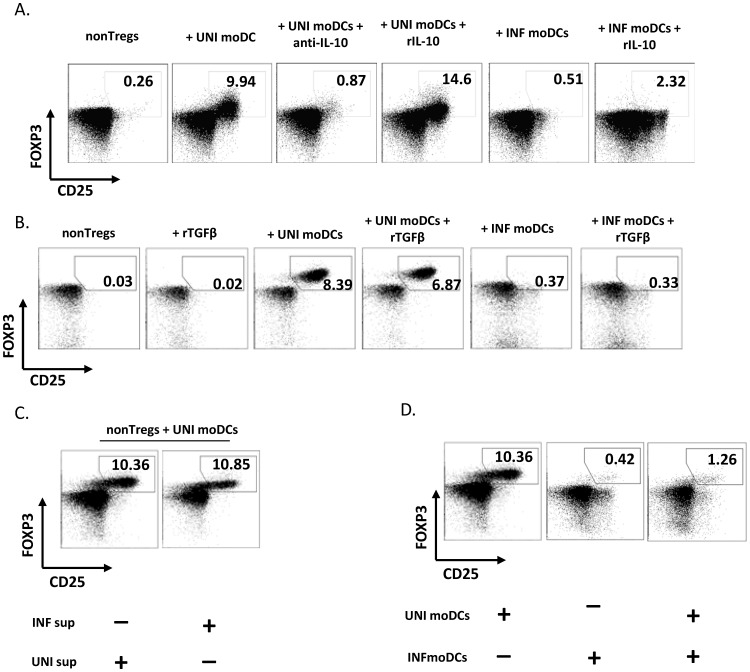
Soluble factors are not responsible for the lack of Treg induction by infected DCs. A) Flow cytometric analysis of the percentage of induced CD25^+^FOXP3^+^ T cells by DC, in the presence of anti-IL-10 Ab (10 µg/ml) or rIL-10 (10 ng/ml). One representative example of 3 independent experiments is shown. B) Flow cytometry analysis of the percentage of induced CD25^+^FOXP3^+^ T cells by DCs, in the presence of rTGF-β(10 ng/ml). One representative example of 4 independent experiments is shown. C) Flow cytometry analysis of the percentage of induced Tregs after culture with uninfected (UNI) DCs for 5 days, in presence of DC supernatants collected from cultures containing either uninfected or infected DCs (6 hours post infection). D) In the same experiment, nonTregs were either cultured at 10∶1 ratio with uninfected or infected moDCs or they were mixed at the 10∶0.5∶0.5 ratio (nonTregs : infected moDCs : uninfected moDCs). One representative example of 3 independent experiments is shown.

Almost 50% of converted CD25^+^FOXP3^+^ Tregs expressed GARP and Cytotoxic T lymphocyte antigen-4 (CTLA-4), markers associated with Treg function [35], [36], whereas CD25^−^FOXP3^−^ cells present in the same cultures express these markers at low levels (<6%, p = 0.006 and <2%, p<0.001, paired t-tests, respectively) (Figure S4). The majority of converted CD25^+^FOXP3^+^ cells proliferated *in vitro,* as assessed by their low levels of CFSE (74.31% ±6.60% were CFSE^low^).

**Figure 6:**
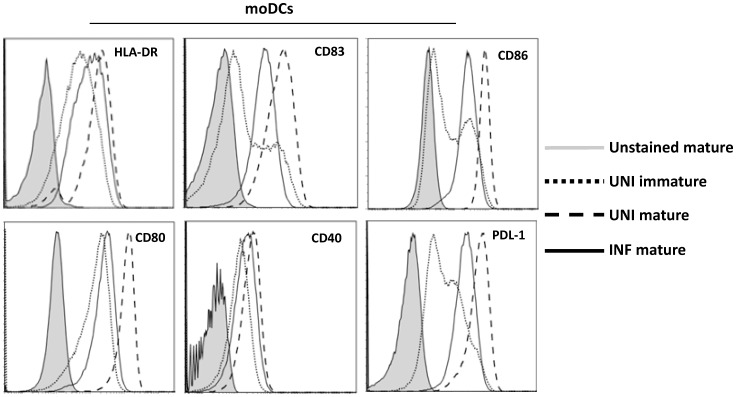
HIV impairs the maturation and activation of moDCs. Expression of maturation, activation and HLA-DR molecules was analyzed by flow cytometry in uninfected immature (dotted line), *in vitro* infected over night mature (solid black line) and uninfected mature moDCs (dashed line). Filled histograms represent the level of expression of each marker by unstained mature moDCs. One representative example of 19 experiments is shown.

### Absence of Treg Conversion by Infected DCs is not Due to a Soluble Factor

To investigate the mechanisms responsible for the lack of Treg induction in infected co-cultures, we examined whether this inhibition involved soluble factors. We first focused on IL-10 and TGF-β because these cytokines play a critical role in Treg induction. Treatment with anti-IL-10 completely blocked Treg conversion in uninfected cultures, whereas addition of rIL-10 increased Treg conversion in these cultures. However, addition of rIL-10 did not restore Treg conversion in infected co-cultures (Figure 5A), ruling out that low IL-10 production could be an underlying mechanism. rIL-10 enhanced the expression of CD25 in FOXP3^−^ cells in infected co-cultures (Figure 5A). These CD25^+^FOXP3^−^ cells did not divide (data not shown), in line with previous reports showing that exogenous IL-10 induces a population of anergized T cells [37], [38], which fail to suppress normal CD4^+^ T cell proliferation *in vitro* [39].

**Figure 7:**
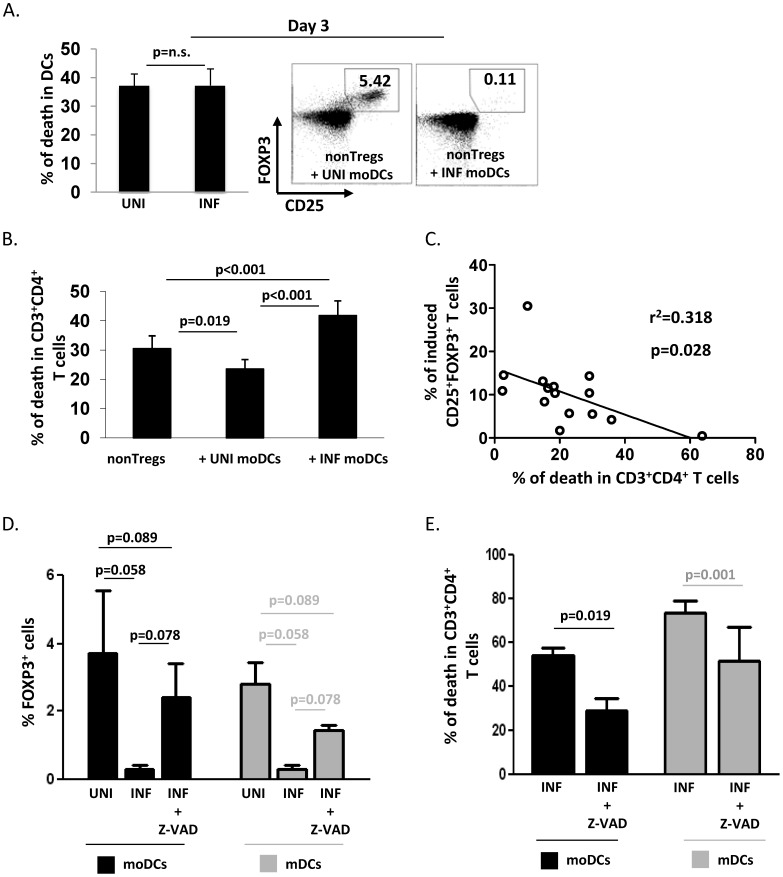
DC-mediated Treg conversion is partially restored in infected co-cultures by inhibition of apoptosis. (A) Percentage of dead DCs was analyzed by flow cytometry using an amine reactive dye (LIVE/DEAD kit, Invitrogen). Graph shows the mean % ± SE of dead CFSE^−^CD3^−^ cells in co-cultures after 3 days and representative dot plots of converted Tregs after 3 days of both uninfected and infected co-cultures are shown (3 independent experiments, paired t-test). B) Percentage of dead T cells was analyzed by flow cytometry using an amine reactive dye (LIVE/DEAD kit, Invitrogen). Graph shows the mean % ± SE of dead CD3^+^CD4^+^ T cells in moDCs-nonTregs co-cultures (19 experiments, paired t-test). C) Percentage of induced Tregs and percentage of dead T cells were correlated in each experiment, using linear regression analysis. D) Mature moDCs or purified mDCs were co-cultured with autologous purified nonTregs in presence or not of the pan-caspase inhibitor Z-VAD (50 µM). The inhibitor was replaced twice a day for 5 days. The graph represents the mean % ± SE of converted FOXP3^+^ cells in uninfected untreated co-cultures or infected Z-VAD-treated co-cultures for both moDCs (n = 4, paired t-test) and mDCs (n = 3, paired t test). E) Mean % ± SE of dead CD3^+^CD4^+^ T cells in infected untreated or Z-VAD treated co-cultures for both moDCs (n = 4, paired t-test) and mDCs (n = 3, paired t test) is shown.

Similarly to IL-10, levels of TGF-β did not appear to be limiting, as addition of rTGF-β did not affect the proportion of converted Tregs in either uninfected or infected co-cultures (Figure 5B). Moreover, HIV infection did not change TGF-β mRNA levels in DCs (mean ± SE: 1.01±0.18 arbitrary units vs. 1.54±0.32 in uninfected vs. infected moDCs, respectively; p = 0.104, n = 6).

To get a broader picture of the involvement of soluble factors, we next tested whether an inhibitory soluble factor was present in DC supernatants from infected cultures. Ruling that mechanism out, our data show that supernatants from infected DCs did not compromise the induction of Tregs by uninfected moDCs (Figure 5C); conversely, supernatants from uninfected DCs did not restore Treg conversion in infected co-cultures (data not shown). Furthermore, in agreement with previous studies [40], separation of DCs and nonTregs by a membrane completely abolished Treg conversion in uninfected co-cultures (data not shown), confirming the necessity of physical contact. Notably, the percentage of FOXP3^+^ cells decreased dramatically when uninfected moDCs were mixed with *in vitro* infected moDCs at a 1∶1 ratio (Figure 5D).

### *In vitro* HIV Infection Alters moDC Phenotype

Impaired Treg conversion could be due to the altered expression of maturation and costimulatory molecules by infected moDCs [1] or primary myeloid DCs [41]–[43]. In agreement with these previous data, *in vitro* HIV-infected and exposed moDCs expressed lower levels of costimulatory and maturation molecules than uninfected mature moDCs (Figure 6). The expression of these molecules was consistently higher on *in vitro* HIV-infected and exposed moDCs than on unstimulated uninfected DCs, while their capacity to induce FOXP3^+^ cells was consistently lower than that of unstimulated uninfected DCs, suggesting that the levels of costimulatory molecules did not affect the capacity to induce Tregs. The inhibitory costimulatory molecule programmed death ligand-1 (PDL-1) has been shown in some models to induce Tregs [44]. As infected DCs expressed lower levels of PD-L1 than uninfected DCs, we directly tested this mechanism, but the addition of rPDL-1 did not restore Treg conversion in infected cultures (mean ± SE % of converted CD25^+^FOXP3^+^ cells was 0.25%±0.09% in infected co-cultures and 0.07%±0.01% after addition of rPDL-1; p = 0.29, n = 3).

### Infected moDCs and Primary mDCs Induce Death of Converted Tregs

HIV infection did not increase DC death after 3 days of co-cultures (p = 0.49, Figure 7A), although, by that time, Treg induction by infected DCs was already severely decreased compared to that induced by uninfected DC (Figures 7A). In contrast, in our experimental system using moDCs, T cell death was significantly higher in infected co-cultures than in uninfected ones (p<0.001, Figure 7B). The percentage of induced Tregs in uninfected co-cultures was inversely correlated with the percentage of T cell death (Figure 7C), suggesting that death of induced Treg could be a mechanism for their low percentage. Confirming this hypothesis, treatment of the DC:non-Treg cultures with pan-caspase inhibitor Z-VAD increased Treg conversion (Figure 7D) as well as T cell survival (Figure 7E) in both infected and uninfected co-cultures, thus directly linking Treg death to the decreased conversion observed in HIV-exposed DC cultures. In contrast, addition of exogenous IL-2 in infected co-cultures neither rescued cell viability nor restored Treg conversion (not shown). Of note, these results were confirmed using primary DCs. Z-VAD treatment significantly increased Treg conversion (Figure 7D) and decreased T cell death (Figure 7E), although the effect was partial as the percentage of Treg conversion in Z-VAD treated cultures remained significantly lower than that in uninfected cultures.

## Discussion

DC-T cell contact is crucial for the regulation of immune responses and any perturbation in DC function would result in significant changes in the tolerance/immunity balance. Due to their location at mucosal surfaces and their expression of HIV receptors, DCs are important targets of HIV infection. HIV-infected myeloid DCs exhibit defective maturation even upon strong microbial stimulation [45], [46], a result confirmed by our study, although opposite results were obtained if HIV-1 Bal infected DCs were stimulated by CD40L [47]. How these defects affect the ability of circulating DCs to induce Tregs is poorly understood, and clarifying this issue was the goal of our study.

We show that uninfected LPS-matured moDCs induce a higher proportion of Tregs than immature DCs. LPS is a classical DC stimulus, which we used as a model to examine whether mature DCs could induce Tregs in an autologous system. Of note, similar defects were obtained with DCs activated with gram-positive bacteria, suggesting a broad phenomenon. The finding that bacteria-activated moDCs induce Tregs more efficiently than immature moDCs is in agreement with previous reports [19], [40], [48], although it appears paradoxical because immature DCs classically induce tolerance through the production of regulatory cytokines and induction of Tregs both *in vivo* and *in vitro* [49], [50]. However, an important characteristic of our model is that we used an autologous system without T cell-specific stimulation. In this system, mature DCs could trigger Treg induction as a mechanism of protection against excessive immune responses. Of note, our experiments demonstrated the crucial role of DC-T cell contact in Treg conversion as this process was completely inhibited by separating these two cell populations, in line with previous studies [40], [51], suggesting the involvement of TCR-mediated signals in this pathway. It has been shown that costimulatory molecules on mature DCs are required to maintain self tolerance [52]. Immature DCs may be better at inducing Tr1 cells, IL-10 producing cells that do not express FOXP3 [49].

Importantly, the FOXP3^+^ T cells generated by uninfected moDCs not only expressed markers associated with natural Tregs such as CD25 or CTLA-4 [36], [53] but appear to be functional in suppressing the proliferation of conventional T cells, a result in agreement with previous reports [19], [40]. As FOXP3 expression cannot be used for live cell sorting, we did not formally prove that these CD25^+^FOXP3^+^ cells were suppressive. It is entirely possible that a suppressive factor was present in the bulk cultures; however, this putative factor is closely associated with the presence of CD25^+^FOXP3^+^ cells, as bulk cultures with <1% FOXP3^+^ cells did not suppress the proliferation of responder cells.

In the current study, we found that peripheral blood mature moDCs as well as mDCs induced a lower proportion of Tregs than uninfected DCs. Importantly, similar data were obtained with primary mDCs and moDC, confirming that moDCs constitute a good model to study this particular aspect of myeloid DC biology. However, these new results contrast with our recent data, which showed that tissue isolated mDCs from SIV-chronic infected macaques induced higher levels of Tregs compared to uninfected macaques [21]. A potential explanation for the differences between our two studies could be that mDC-mediated conversion is more or less efficient at different stages of infection. Our *in vitro* system mimics the early phase of the infection, when DCs are exposed to high doses of virus. In line with these observations, extensive Treg death occurs in the highly pathogenic model of acute SIV-infection of pigtailed macaques [54], whereas increased proportions of Tregs are found during chronic HIV/SIV infection [10], [11], [13], [22], although the mechanisms underlying these differences have not been studied in these reports. Thus, it is possible that Treg conversion can occur when viral loads are relatively low, such as in the chronic phase, but is severely impaired during the acute phase [21]. Further studies conducted with *in vivo* differentiated DCs, purified from different tissues of SIV-infected macaques at different stages of infection, will be needed to clarify this important question.

As several pathways are known to be involved in DC-mediated conversion, we studied which pathway(s) could be defective in HIV-infected moDCs. Our results show that the low Treg induction by both HIV-exposed moDCs and primary mDCs, is mainly due to the selective death of induced Tregs in contact with these DCs. Supporting this hypothesis, Treg induction was inversely correlated with T cell death. Moreover, a significant improvement in Treg induction was achieved by blocking caspases. Killing of T cells by HIV-infected DCs has already been reported, but pDCs have been more extensively studied than mDCs [55]. In particular, HIV infection of pDCs activates TRAIL, and these DCs induce apoptosis of CD4^+^ T cells that express the death receptor TRAIL R2 [56]. Interestingly, Lichtner et al. showed that HIV-pulsed moDCs induce apoptosis in approximately 40% of autologous uninfected T cells, via multiple caspase-dependent mechanisms, including FasL, TRAIL, TNF-α and TWEAK [56]. Similarly, Hardy et al. showed that HIV induced expression of TRAIL on pDCs and turned them into killer pDCs [57]. The fact that we could not completely rescue Treg induction by the pan-caspase inhibitor could have several explanations. One could be that the dose we used was not sufficient to achieve full blockade. Alternatively, other mechanisms, such as decreased expression of costimulatory molecules or PDL-1 by infected DCs, could play a minor, but synergistic role with their increased killing of Tregs. One intriguing result in our model is that Tregs appear more susceptible to apoptotic death than nonTregs. Baatar et al. showed that Tregs express high levels of FasL [58], which could constitute a potential mechanism.

Several important factors must also be noted. In the experiment where infected DCs were mixed with uninfected DCs, Treg conversion was not restored. A likely explanation is that there was a sufficient number of infected DCs able to kill converted Tregs. Alternatively, the exposure of uninfected DCs to the infected ones may have altered their physiology and render them unable to induce Tregs.

Of note, selective Treg death was not due to a deficit in cytokines important for Treg survival, as addition of exogenous TGF-β or IL-2 to HIV-infected cultures did not decrease Treg death nor did they restore Treg conversion. Second, Treg death was not due to a virus transfer from DCs to T cells, and their subsequent death due to a direct cytopathic effect of infection. Indeed, similar death and low Treg induction were found when inactivated virus, unable to infect DCs or T cells, were used or when AZT was added to the cultures. Last, our data do not support the hypothesis that increased, and early death of HIV-exposed DC, which would also be controlled by addition of a pan-caspase inhibitor, plays a major role in their defective induction of Treg. Indeed, DC death was similar in uninfected and infected co-cultures at an early stage of co-culture (3 days), when defective induction of Treg was already notable (Figure 7). Taken together, our results suggest that Treg conversion is an early and dynamic event, triggered by contact with mature DC, followed by expansion *in vitro* of these induced Tregs an exposure of DC to HIV profoundly affects this pathway early on.

In summary, our data suggest that death of induced Tregs may play a role during the acute phase of pathogenic infection. As Tregs control HIV replication in several cellular targets such as T cells and macrophages [59], [60], killing of Tregs by DCs exposed to high titers of virus could hamper their capacity to restrain HIV replication in the early phase of infection, and thus could be an important factor in HIV pathogenesis.

## Supporting Information

Figure S1 **Uninfected moDCs do not expand circulating Tregs.** Circulating Tregs (CD4^+^CD25^hi^CD127^low^) or nonTregs (CD4^+^CD25^low^CD127^hi^) were purified by cell sorting, CFSE-labelled, and cultured with autologous LPS-activated uninfected (UNI mature) moDCs for 5 days. One representative flow cytometric analysis (out of 3 independent experiments) of expression of CD25, FOXP3 and CFSE is shown. Numbers represent the percentage of positive cells.
(TIFF)

Figure S2 **LPS stimulation of nonTregs does not induce FOXP3 expression.** Bead-purified nonTregs from PBMCs were left unstimulated (grey lines), or cultured with LPS overnight (dotted line) or for 5 days (black line). FOXP3 expression is shown for one representative example for each group (n = 3/group).
(TIFF)

Figure S3 **VLP SIVmac alone does not block DC-mediated Treg conversion.** In 4 independent experiments, LPS-stimulated moDCs were infected with VLP SIVmac alone (MOI = 3), or left uninfected, before co-culture with autologous nonTregs. Percentage of CD25^+^FOXP3^+^ T cells was analyzed 5 days later. One representative experiment is shown.
(TIF)

Figure S4 **Converted Tregs express GARP as well as CTLA-4.** Mean ± SE % of GARP^+^ or CTLA-4^+^ cells in converted CD25^+^FOXP3^+^ Tregs and CD25^−^FOXP3^−^ T cells is shown in A (n = 5) and B (n = 6), respectively. P values correspond to paired t-tests.
(TIFF)
