# Supplementary figures and images for: In Vitro HIV Infection Impairs the Capacity of Myeloid Dendritic Cells to Induce Regulatory T Cells
Source: PLoS One. 2012 Aug 13;7(8):e42802. doi: 10.1371/journal.pone.0042802 (PMC3418294; doi:10.1371/journal.pone.0042802)

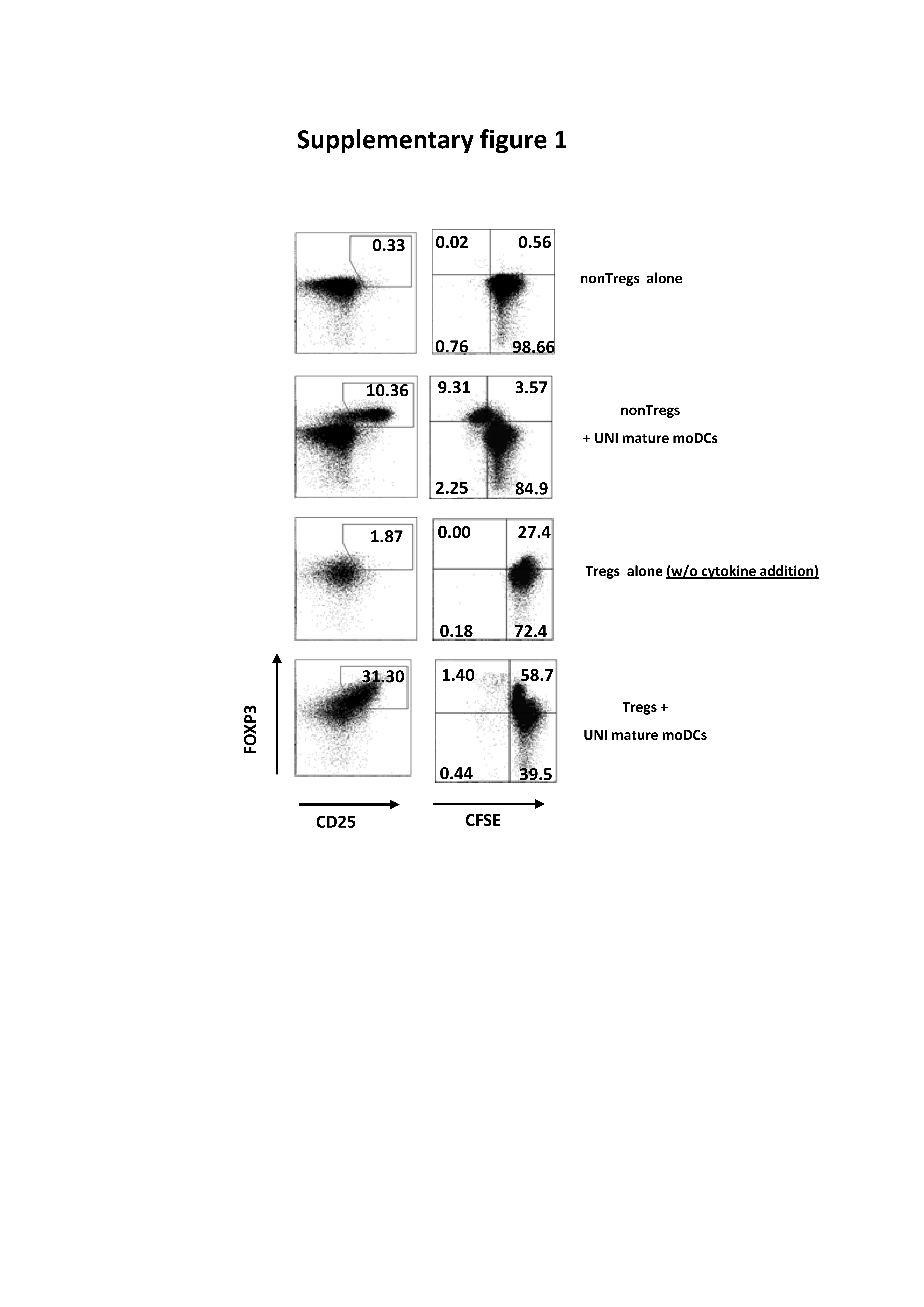

Supplement: Figure S1 — Uninfected moDCs do not expand circulating Tregs. Circulating Tregs (CD4+CD25hiCD127low) or nonTregs (CD4+CD25lowCD127hi) were purified by cell sorting, CFSE-labelled, and cultured with autologous LPS-activated uninfected (UNI mature) moDCs for 5 days. One representative flow cytometric analysis (out of 3 independent experiments) of expression of CD25, FOXP3 and CFSE is shown. Numbers represent the percentage of positive cells. (TIFF) [file pone.0042802.s001.tiff]

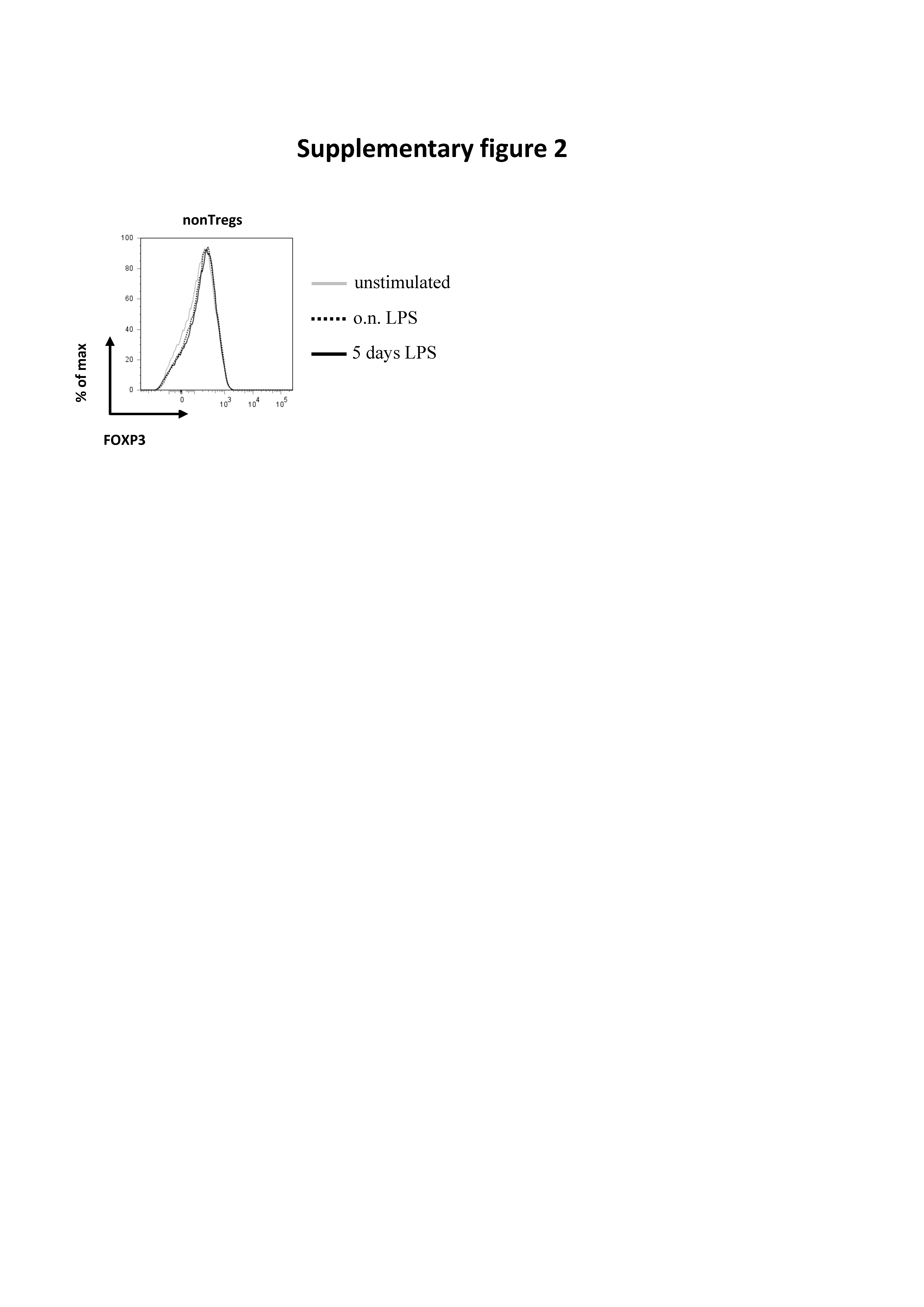

Supplement: Figure S2 — LPS stimulation of nonTregs does not induce FOXP3 expression. Bead-purified nonTregs from PBMCs were left unstimulated (grey lines), or cultured with LPS overnight (dotted line) or for 5 days (black line). FOXP3 expression is shown for one representative example for each group (n = 3/group). (TIFF) [file pone.0042802.s002.tiff]

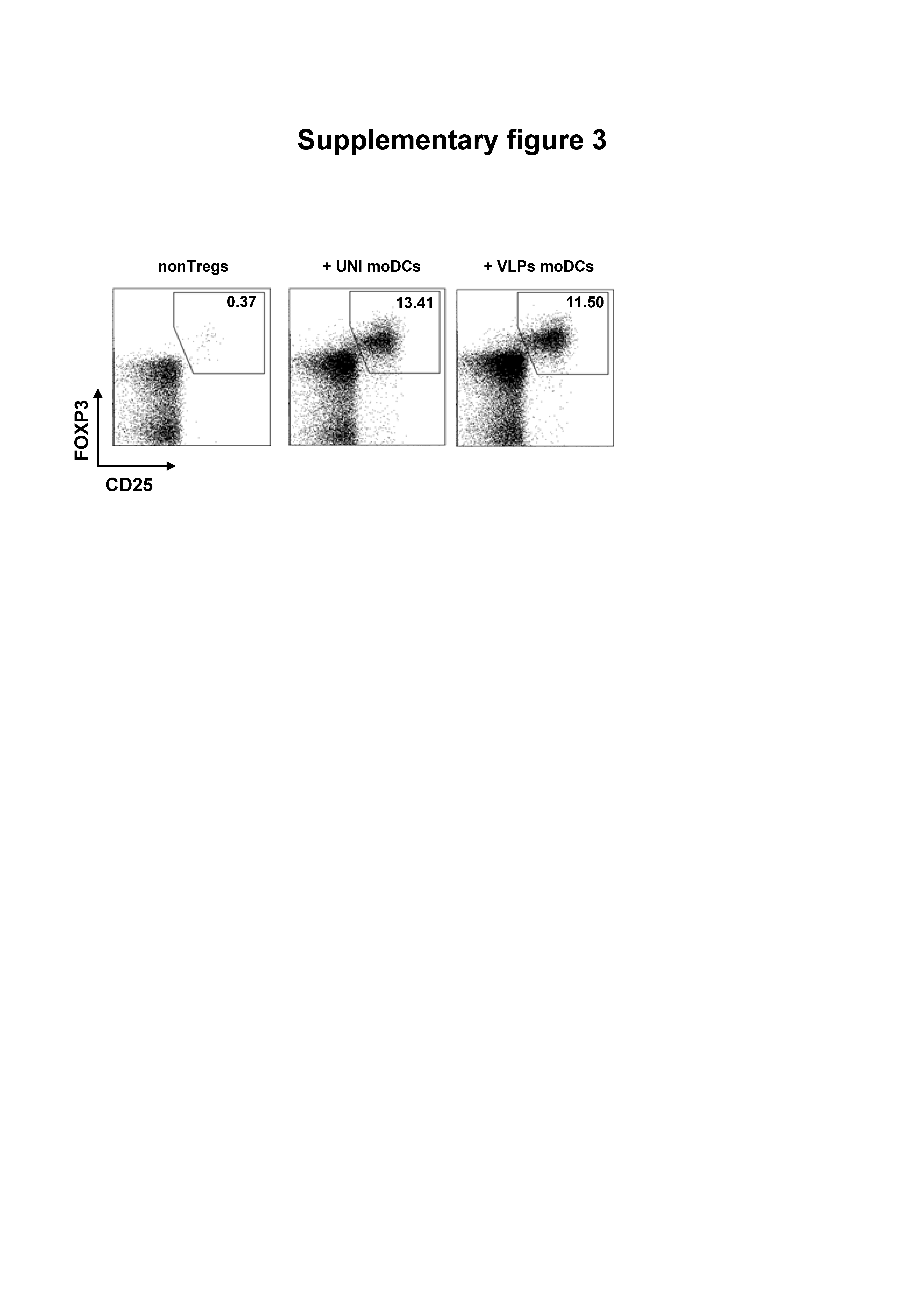

Supplement: Figure S3 — VLP SIVmac alone does not block DC-mediated Treg conversion. In 4 independent experiments, LPS-stimulated moDCs were infected with VLP SIVmac alone (MOI = 3), or left uninfected, before co-culture with autologous nonTregs. Percentage of CD25+FOXP3+ T cells was analyzed 5 days later. One representative experiment is shown. (TIF) [file pone.0042802.s003.tif]

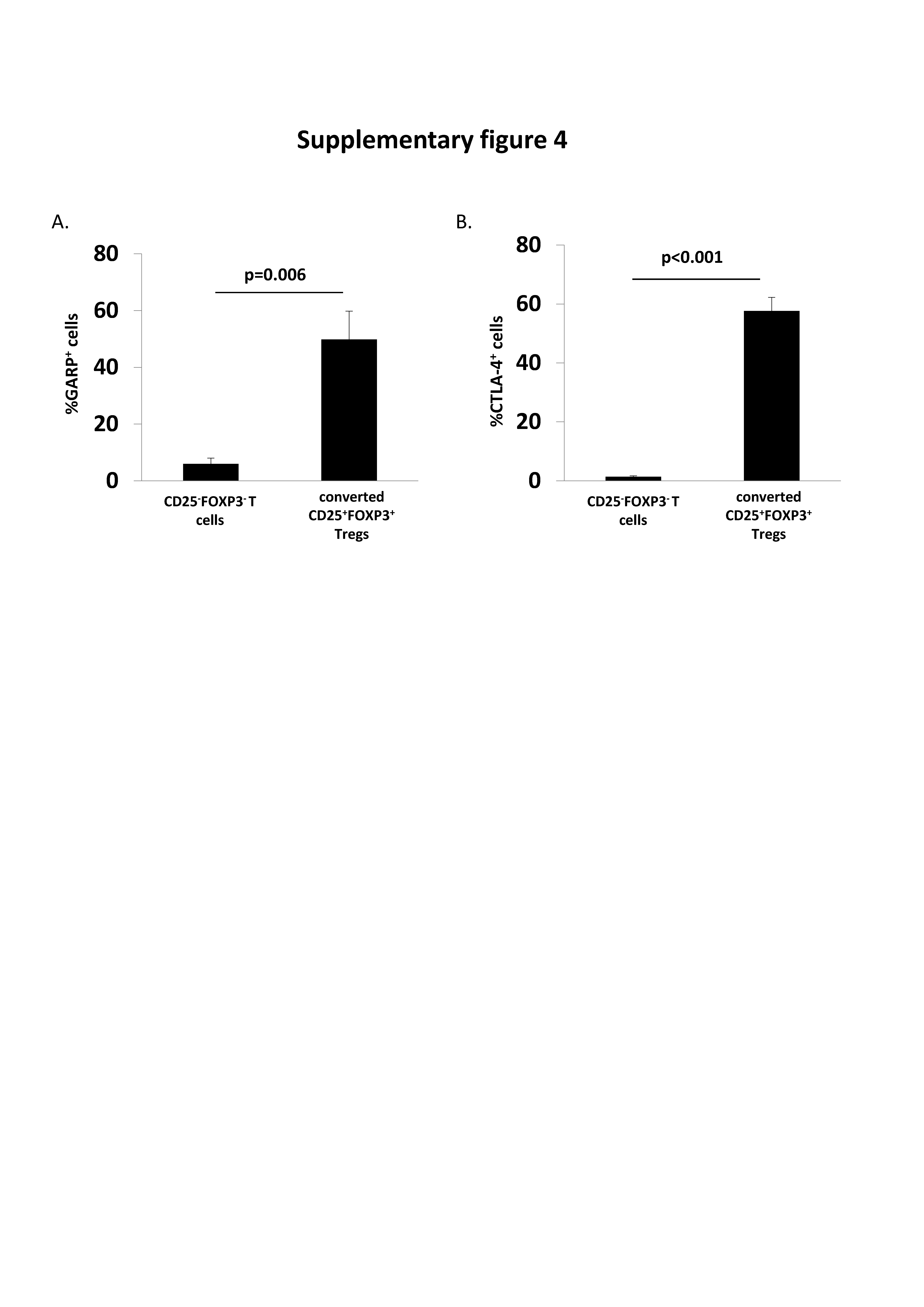

Supplement: Figure S4 — Converted Tregs express GARP as well as CTLA-4. Mean ± SE % of GARP+ or CTLA-4+ cells in converted CD25+FOXP3+ Tregs and CD25−FOXP3− T cells is shown in A (n = 5) and B (n = 6), respectively. P values correspond to paired t-tests. (TIFF) [file pone.0042802.s004.tiff]
